# Facilitators and barriers to contraceptive uptake and use: Perspectives of female students in South Africa and lessons for quality provision of HIV pre-exposure prophylaxis

**DOI:** 10.1371/journal.pone.0333270

**Published:** 2025-09-24

**Authors:** Patience Shamu, Nicola J. Christofides, Saiqa Mullick

**Affiliations:** 1 Wits RHI, University of the Witwatersrand, Johannesburg, South Africa; 2 School of Public Health, Faculty of Health Sciences, University of the Witwatersrand, Johannesburg, South Africa; The University of Texas at San Antonio School of Public Health, UNITED STATES OF AMERICA

## Abstract

Young women in South Africa face a dual risk of unplanned pregnancies and HIV infections. The country’s high contraceptive prevalence rate masks underlying challenges affecting the initiation and maintenance of contraceptive use. Options are freely available at public health clinics and most women in the country use long-acting injectable contraceptives. Young women’s experiences of contraceptive services provide a unique opportunity to learn lessons for long-acting HIV Pre-exposure prophylaxis (PrEP). The paper aims to explore facilitators and barriers to using contraceptives among young female students to inform the delivery of long-acting PrEP. A qualitative study was conducted in one district in Gauteng province of South Africa. We purposively selected female students 18–24 years seeking sexual and reproductive health services (SRH) through fixed or mobile clinics in 2022 on two campuses. We conducted 22 in-depth interviews and five Focus Group Discussions (FGDs). Data were thematically analysed using NVivo 12 software. Individual-related barriers were mainly related to the side effects of contraceptives. At a family level, mothers sometimes played a supportive role which enabled continued contraceptive use, yet partners’ concerns about side effects were sometimes a barrier. Health systems-related issues included negative health provider attitudes and unfavourable opening hours. Accessing contraceptives over holidays when students travelled was a challenge. Long-acting injectable PrEP may be compatible with injectable contraceptives and could provide an opportunity to align visits. Training service providers on the importance of detailed information on side effects will be crucial. Promoting PrEP among mothers and partners could increase support for continued PrEP use. These lessons can be applied to delivering new long-acting HIV PrEP products near the market.

## Introduction

Many young women face a dual risk of HIV infection and unintended pregnancies. The combined risks pose a challenge to the achievement of the United Nations Sustainable Development Goals for 2030. Adolescent girls and young women (AGYW) aged 15–24 years, especially in sub-Saharan Africa (SSA), are disproportionately affected by HIV, accounting for nearly two-thirds of new infections in 2021 [1]. A national survey conducted in South Africa reported that the HIV prevalence in this age group was 10.9%, substantially higher than that of their male counterparts (4.8%) [2]. Also, the annual incidence of HIV among AGYW was high at 1.51% compared to 0.5% among men [2]. Despite the availability of many different HIV prevention interventions, such as male and female condoms and voluntary male medical circumcision, young women continue to be disproportionately affected by HIV as these methods are mainly male-controlled [3], leaving women with little control over HIV prevention. Pre-exposure prophylaxis (PrEP) is being rolled out as part of HIV prevention interventions as it gives women power over HIV prevention. Oral PrEP became available in South Africa in June 2016 for female sex workers and the following year for men who have sex with men [4]. Access to oral PrEP has now been expanded to everyone considered at substantial risk of HIV through primary healthcare (PHC) facilities. Long-acting PrEP formulations such as the Dapivirine Vaginal Ring (DVR) are now available through selected pilot sites [5]. The injectable cabotegravir (CAB-LA) has been approved by the South African Health Products Regulatory Authority (SAHPRA) [6].

The most recent Demographic and Health Survey in South Africa shows that the contraceptive prevalence rate among sexually active women, aged 15–49 years, was 60%. Married women and those in unions had lower contraceptive prevalence (55%) [7]. These prevalence rates, based on a cross-sectional household survey, do not capture contraceptive use over time. Women may discontinue use when they decide to become pregnant or for other reasons and then use contraceptives again later. Hormonal contraceptives, especially injectables, are most commonly used and account for 25% among sexually active women [7]. The high CPR among sexually active women in the country masks underlying challenges in the quality of care and inequitable access, which affects users’ correct and consistent use [8]. Studies report that quality of care is integral to uptake and continued use of service [9]. In addition, provider attitudes and the availability of contraceptives influence young women’s decisions to use contraception.

Among young women in South Africa who had a pregnancy under the age of 20 years, nearly two-thirds (64%) described it as unplanned and 15% as unwanted [7]. This indicates that there is an unmet need for contraception in this age group [7]. Access to contraceptive services remains a challenge despite the existence of an enabling policy that includes termination of pregnancy [10]. Quality of contraceptive services remains a concern, especially for young women [8]. Service providers’ hostile attitudes towards adolescents and young women discourages utilisation of services [11].

Women who are not using a barrier method (16% prevalence) in addition to hormonal contraception are at risk of HIV. PrEP is a method of preventing HIV using antiretroviral drugs and is considered to have enormous potential in countries with a high-prevalence setting, like South Africa. PrEP grants users, especially women,-control over HIV prevention and is considered vital in settings where gender inequality continues [12]. It is therefore important to draw lessons from contraception services and apply them to the delivery of PrEP. There are challenges related to the acceptance of PrEP, for example implementers highlighted how it was disapproved by the community, especially among parents and religious leaders as it was perceived to encourage AGYW to be sexually active, take risks and to have multiple sexual partners [13]. In addition, healthcare providers have also raised concerns that stigma and lack of PrEP awareness among communities negatively affect PrEP uptake and use especially among the adolescent girls [14].

Like contraceptives, expanding choices for HIV prevention is important. However, expanding choices alone without considering how services are delivered may fail to improve the uptake of services and may not address the persistent unmet need. Previous studies have suggested that the quality of service, provider attitudes and availability of contraceptives influence young women’s decisions to take contraception [15,16]. There is still a need to understand the challenges faced by young women with the use of current contraceptive offerings and apply those lessons to the rollout of new long-acting PrEP methods, especially if the service delivery is to be integrated. We focused on young women aged 18–24 years, as they are at greatest risk of HIV. Students are a priority group as the university environment compounds students’ vulnerability as it brings new freedoms likely to result in sexual experimentation [5], including having multiple partners and transactional sex, especially for first-year students who may be leaving home for the first time [17]. This paper, therefore, aims to explore the barriers and facilitators of the use and delivery of contraceptives among female students to inform the delivery of long-acting PrEP formulations.

## Methods

### Study design

We conducted an exploratory, formative qualitative study between 11 August 2022 and 15 March 2023 to identify barriers to using current contraceptive offerings among students to inform the delivery of long-acting PrEP formulations. The study followed the Consolidated criteria for Reporting Qualitative research (COREQ) guidelines [18]. Using the socio-ecological model (SEM), we framed questions on young women’s experiences using contraceptive and HIV prevention methods. SEM was selected as it the best for understanding complex, multifaceted issues influencing health outcomes as it acknowledges the interconnectedness of different systems. The model categorises the different factors influencing young women’s access and use of contraception into individual, household, organisational, community and policy levels [19]. Understanding facilitators and barriers to service utilisation using a multifaceted approach is crucial in informing long-acting PrEP delivery.

SEM was selected as it allows for the understanding of complex, multifaceted influences on health outcomes which is crucial in informing long-acting PrEP delivery. While the SEM was the best for this study, the application of the model had limitations as the study was conducted with young women only and their perceptions of the relationship, family and health service level influences on their contraceptive use. Understanding factors from other perspectives, e.g., healthcare providers, could have been valuable. In addition, participants did not engage with sociopolitical (macro-level) factors which is an important part of the SEM. As long-acting PrEP methods become available, these factors, including the service delivery attributes, may influence students’ decisions to use the methods. The methodology for this study was previously described [20]

### Study setting

The study was conducted in the City of Tshwane, South Africa’s Gauteng Province. Of the eight metropolitan areas in South Africa, the City of Tshwane has the fourth biggest HIV prevalence among young women aged 15–24 years at 10% [7]. The district was purposively selected as it is part of the She Conquers 22 high-priority districts currently providing comprehensive SRH services through public sector facilities. She Conquers is a multi-sectoral South African government-driven campaign aimed at reducing new HIV infections in girls and young women, reducing teenage pregnancies, reducing sexual and gender-based violence, and increasing economic opportunities for young people [21]. Two tertiary institutions, a university and a technical vocational college were purposively selected for the study. The two sites frequently get services from mobile clinics which visit the institutions on certain days of the week. These mobile clinics are managed through a government-run Community Health Centre (CHC). Mobile clinics provide SRH services such as screening and testing for sexually transmitted infections, HIV testing, counselling, making referrals for clients who test positive, and contraceptive services, including oral PrEP for HIV prevention. The services are free of charge to users and are financed through external donor funding supporting the South African government.

### Population and sample

Young women aged 18–24 years were purposively selected from campus and mobile health clinics where they were accessing similar SRH services. They were selected based on having current or recent experience of contraceptive use. The campus clinic provides similar SRHR offered by mobile services, but they also offer treatment for minor ailments.

### Data collection procedures

Trained female field workers experienced in qualitative data collection recruited participants who were aged 18–24 and had used contraceptives. We trained field workers on research ethics, questionnaire administration and field work logistics. Role playing was used to assess comprehension of the data collection procedures. Information about the study was provided to students in the university’s campus clinic’s waiting area, mobile clinic or from the college campus, and students volunteered to participate. An informed consent process was carried out with individual volunteers. We conducted face-to-face In-Depth Interviews (IDIs) and FGDs in English as the participants spoke diverse languages; however, if participants wanted to express some things in a vernacular language, they were encouraged to do so as the data collectors were multilingual. PS and the field workers field-tested the tools. The field workers both had an honours degree in social studies and were both young women, while PS had a master’s degree and also worked as a researcher. Semi-structured interview guides were used for both IDIs and FGDs. FGDs were used to capture students’ perceptions of new long-acting PrEP technologies while IDIs explored their experiences of using either contraceptive methods or HIV prevention methods. We conducted IDIs in a boardroom or outdoors with auditory privacy on both campuses. The IDIs took 30–60 minutes and were audio-recorded. Field workers captured field notes both during and after the IDIs.

The FGDs had 6–10 participants. We recruited participants through the same places as described for the IDIs. Students who were interested in participating in FGDs approached the field workers to provide details of their [22] availability and the interviews were scheduled around the student’s availability. The FGDs were 1.5–2 hours long and were audio-recorded. The note taker for each FGD captured notes during the interview.

### Ethics approval and consent to participate

This study was performed in line with the principles of the Declaration of Helsinki. Clearance to conduct the study was obtained from the University of the Witwatersrand Human Research Ethics Committee [Medical] [protocol number [M2111133]. Permission was obtained from the institutions’ management to conduct the study on campus. We obtained written informed consent from participants for the participation of the IDIs and FGDs and for the audio recordings. Participants received R50 [USD2.9] for participating to reimburse them for the time that they spent in the study.

### Data management and analysis

Data were audio recorded and transcribed verbatim. Two field workers checked the transcripts to ensure the excellent quality of the documents before sending them to the researcher for final checking. All IDI and FGD data were coded using NVivo 12 software (QSR International)

During data analysis, PS worked as a qualitative researcher in a PrEP study, which provided additional insights. We adopted a thematic approach to data analysis. Both inductive codes, identified through a deep reading of the transcripts and deductive codes based on the objectives and the literature were developed [22]. To maintain rigor in line by line analysis of the transcripts, we ensured a transparent coding process through maintaining an audit trail by writing detailed memos. All transcripts were coded, and during the process, some codes were merged while a few new codes were added. The socio-ecological model was kept in mind during the development of these codes. These codes were then refined inductively. We then generated initial themes and reviewed potential themes based on patterns in the data. Themes and codes were discussed with the co-authors until an agreement was reached, and we finally produced a report.

### Findings

Twenty-two young women were selected for IDIs, and 43 participated in five FGDs. We reached saturation on students’ experiences of contraceptive and HIV prevention methods use.

As depicted in Table 1, most IDI participants grew up in the Gauteng province. The average age of participants was 21 years. Most of the participants were using injectable contraceptives, Nur-Isterate and Depo Provera. Most of the participants had never used PrEP. Six of the IDI participants had been pregnant before, and five had children. The sociodemographic characteristics of FGD participants were similar to those who participated in the interviews, with an age range of 19–24 years. The college students stayed off-campus in rented accommodation in the township where the college was located as the college did not provide accommodation. Most university students were staying on campus while a few others were staying with parents, friends or by themselves.

**Table 1:** IDI Participants’ demographic characteristics.

Variable	Level	Frequency (n = 22)	Percentage (%)
**Participant age (years)**	19	5	23
	20	3	14
	21	5	23
	22	4	18
	23	4	18
	24	1	4.5
**History of pregnancy**	Yes	6	27
	No	16	73
**Parental status (Have children)**	Yes	5	23
	No	17	77
**Home Province**	Limpopo	7	32
	Mpumalanga	5	23
	Kwa-Zulu Natal	1	4
	Gauteng	9	41
	Eastern Cape	0	0
**Type of contraception used**	Pills	4	18
	Nur-Isterate	7	32
	Depo Prover	7	32
	Implant	1	4
	Discontinued use	3	14
**Childhood residence**	Rural	11	50
	Urban	9	40
	Peri-urban	1	5
	No response	1	5
**Previous use of PrEP**	Yes	5	22.7
	No	14	63.6
	No response	3	13
**Total**		**22**	**100**

Participants’ experiences were categorised into two main themes: facilitators and barriers, with sub-themes encompassing the different levels of the social-ecological framework including individual, relationship, family, community, and health system-related factors. Facilitators were described as factors that enabled the continuous utilisation of contraceptive services in this study while barriers were the factors that hindered the uptake and/ or continued use of contraception. Most of the facilitators or barriers were health system-related.

### Facilitating factors

#### Individual level factors.

The main facilitating factors for initiation or continued use of contraceptives at the individual level were satisfaction with the type of contraception, the perceived benefits as well as the young woman’s agency to use contraception regardless of partner support. Some participants described that their goal to complete studies enabled them to exercise their agency and make decisions that would support achieving their goals.

*“I would do my studies and I would know what I’m getting myself into, so, no matter his response, I’m the one that will be left to make the decision [about contraceptives” [* **IDI Participant 03-01-05, 20-years]**

The perceived benefits of using contraception cited by participants included preventing pregnancy; and using an injectable contraceptive meant only having to return to the health facility every two months. Also, contraceptive methods which provided additional off-label benefits included having a clear skin that was free of acne and pimples.

*“Until now, I can see that the benefits were that acne was less, compared to now… I also saw that they were also benefiting me in another way that I didn’t expect… the positive side is that they helped me to prevent acne, and at the same time they also helped to prevent not being pregnant and all you know, yeah.” [* **IDI participant 01-01-12, 22-years** *]*

Young women also reported other perceived benefits of using contraceptives. These included having hormonal balance as they said they believed that they suffered from hormonal imbalances before using contraception. Other participants were satisfied with gaining weight as they used the contraceptives. A participant said, “*My experience about Nuri [*Nuristerate*] ne, ahh Nuri was treating me so fine because I gained weight, which was good to gain weight”* **[IDI participant, 01-01-06, 19 years]**

#### Relationship and family factors.

Partner and family support enabled the continued use of contraceptive services. Partner support included reminding participants to go for their next appointment or accompanying them to the health facility.

“*…my partner supports me, and he makes sure that I don’t skip, he even accompanies me here, so yeah”* **[Participant P27, FGD 03-01-01].**

A student explained how having clear goals and sharing them with her partner enabled continued use of a contraceptive method. She explained,

*“…for me, my partner knows that I am preventing pregnancy like this I explained to him that I want to get somewhere in life where I would feel comfortable to have a child. I don’t want to have child whereby it will be a burden, my child and I are going to be a burden to my mother and then I am being raised by a single parent so I don’t want that for myself.”* (**FGD01-01-03, P23).**

Getting family support from mothers supported continued contraceptive use. Mothers played a key role in providing information on the methods suitable for their daughters. In addition, some mothers played a monitoring role checking that the participants had not missed their appointments to get their contraceptives. A few mothers provided practical support either by providing money or driving their daughters to the health facilities.

“…*when my mother has money she’ll give me to go to the clinic…to the doctor, but if let’s say I got injected 2 months back then they give me a return date and I’m home, then I’ll be able to go to the clinic, but if I skipped my return date like now, I didn’t get injected then she will give me money to go to the doctor. When I get to the doctor I buy a pregnancy test to show them that I am not pregnant then I get my injection.” [***IDI participant 01-01-06, 19 years].**

Mothers also played an advisory role in contraceptive method choices based on their beliefs and perceptions about the different methods. A participant explained how her mother advised her on which contraceptive method to use:

“*I started my contraceptives with depo, so my mother told me that, no, usually depo is used by women who already have children because it makes you fertile. So, when you stop injecting, like using the injections, you immediately after 6 months fall pregnant. It’s best I use the two months one because I’m still young, and I’m not in the process of starting a family anytime soon.”* ***[*Participant 23, FGD01-01-03].**

#### Heath service factors.

Positive provider attitudes facilitated contraceptive service use continuation. Positive attitudes were mainly reported by students accessing services through mobile and campus clinics which participants perceived were very different from those at PHCs. In FGDs, students agreed that providers at mobile and campus clinics had positive attitudes, which enabled regular access to the services. They explained that the providers were approachable and that the students could share personal issues with them. They also reported being satisfied with how nurses counselled them on the potential side effects of contraceptives.

“*they are perfect you communicate when you have uhm, personal things you speak with them they interact everything is good they are perfect, they are good.”* [**Participant P17, FGD 01-01-01].**

Good service delivery for students was about being spoken to respectfully in a non-judgemental way, ensuring privacy, confidentiality, and having short waiting times before the consultation. Some participants spoke about the encouragement to prevent pregnancy they received from providers who stressed the importance of preventing pregnancy when they were not ready for it.

*“The services are great because they encourage you that hmmm…it’s better to prevent, than for you to uhmm…bring a child into this [world], having to uhmm…go through all the emotions of having a child, being a mother when you’re not ready while you’re still a child yourself basically, you see so they just say that, it helps decrease the chances of teenage pregnancy, yeah* **.” [IDI Participant 03-01-05, 20 years]**

The way services were organised contributed to the continued use of services. Providing privacy to clients through separating family planning clients from others was another important factor.

### Barriers to the use of contraceptive services

#### Individual level factors.

**Side Effects.** Most students reported experiencing various side effects as they used contraception. These were mainly related to their menstrual cycle, such as irregular periods or heavy bleeding. Some participants also reported amenorrhea. Some young women saw it as a problem.

“...*I think it’s been almost four months I haven’t been to my periods and then I also used the, the injection, the two months’ injection and it was going well I didn’t have anything, there was nothing wrong that, it worked very well, it worked very well with me.”* **(P15, FGD participant]**

While some participants were happy with weight gain, it was a concern for others. When other side effects accompanied it, it led participants to change methods or discontinue contraception.

“…*and my experience was quite bad with the two-month injection, I had too many side effects, gained weight, I even had a rash at one point, which led to a yeast infection of some sort. So, now I’m considering to actually take uhm…pills instead of the injection, maybe they will work better for me*” **[P9, FGD participant].**

Participants also reported experiencing headaches which led to the discontinuation of contraceptive methods as they disturbed studies. A participant said*, “but some days you felt like the headache was intense so you like hmmm I can’t cope with stuff like this while I have to study, so I was like I am not going to continue with this*” **[01-01-14, IDI participant, 23-years].** In addition to experiencing headaches, some participants also reported having mood swings due to contraceptive use. A participant said, “…*and then it’s also a thing of my mood swings, my mood would escalate or be low and then also hmmm…I had constant headaches*” **[IDI participant 01-01-14, 23 years].** Headaches were a reason for method discontinuation.

Fear of infertility was another perceived side effect reported by some participants. The participants feared that they might end up being infertile because of experiencing heavy bleeding*.* A participant reported that*, “It was too much. I thought I was losing too much blood or something… and I’ll end up not having kids”* **[03-01-02, IDI participant, 23 years]**. Other participants experienced prolonged periods, which took almost a month. A participant explained, “… *I once used uhm…implant and then… maybe I would have my periods maybe for 25 days*” **[P19, FGD01-01-01 participant].**

In addition, some participants reported that they continued experiencing various side effects despite trying different methods and ended up discontinuing contraceptive use. One participant expressed frustration with the current contraceptive offerings after almost exhausting all the available options:

*“I’ve tried almost all the contraceptives because I think eish I don’t, I don’t get to get the contraceptive that actually works for me, I have tried implant it was causing headaches, nausea, excessive appetite and I’ve tried both injections. I was always on my periods with both of the injections and then I tried the pills. I would get an infection in my vagina it would cause me watery discharge and UTIs.”* **[P11, FGD Participant].**

The participant then opted for an intrauterine device (IUD), but the doctor advised her that she could not get it as she was already struggling with infections and urinary tract infections (UTI), and the loop was going to worsen the problem.

#### Struggling with adherence.

Taking pills was difficult for some participants who were concerned that they would forget to take them. Being forgetful was reported to lead to the failure to take contraception.

*“I was scared to use contraceptives because my period they are not regular periods, so I went to the clinic and they, they opted for the oracons and say they would help regulate my cycle. So I started using them and yeah I could see my period when I’m taking the red pills and then now my problem was that eish I’m forgetful…*” **[P14, FGD participant]**

#### Experience with health services.

Previous negative experiences with the health system deterred students’ future use of the services. Negative experiences at health facilities, such as being made to join the same queue as sick people, made some students stop going to some PHCs to get their contraception. In addition, the participants complained about spending long waiting times. A FGD participant reported that,

*“I stopped going to the local clinic, on recess, because when I went there I have to queue with the people who are sick…. like maybe I will go there around 9 and then now I have to leave the clinic like around 5, let’s say knock-off hours, and I’m not going there for, you know like I’m not sick. I’m just here for a simple thing, it won’t even take five minutes….”* **[FGD Participant, P27].**

#### Communication with healthcare providers.

Language barriers hindered some students from accessing reproductive health services. Students coming from other provinces where different languages were spoken were struggling and some ended up deciding not to access the PHCs. One participant said,

*“At school, no, I have never been to the clinic at school I can only tell you about the service where, where at home where I’m coming from because even here it’s difficult for me to, to understand because they speak their language and I don’t understand their language but the one, the language that I understand from back at home.”* **[IDI 3-01-09 participant, 21-years]**

#### Not having a sexual partner.

Not having a sexual partner resulted in some students discontinuing using a contraceptive method. Some students reported that they stopped using contraceptive services during vacation as they were mainly sexually active when they were back on college or university campuses. A participant explained,

*“The time when I was using nur-isterate ne, whenever I went home, I stopped using it because I did not see the need for using it, because I’m home. When I’m home, I’m home, okay. There is no boyfriend. Yeah, remember he stays in Joburg [Johannesburg]”* **[Participant P26, FGD 01-01-01].**

### Relationship and family factors

#### Partner influence.

Male partners were potential barriers to contraceptive use. Some participants reported how their partners discouraged them from continuing to use contraception, especially when they were experiencing some side effects. The partners had misconceptions fearing that the methods would affect their partner’s fertility in future.

“…*he said I must stop using them because they are not right and I’m not having my period at all, what if uhm…after some years when we wanna [want to] have a baby I don’t get a baby because the contraceptives have done something to my body as I’m not going to my periods at all”* **[Participant P14, FGD 01-01-01].**

#### Mothers’ influence.

Participants reported how their mothers played a key role in influencing their decisions to use contraception. Many mothers provided advice and encouragement; however, a few mothers did not want their daughters to use contraception as they were expecting grandchildren. A participant said, “*yeah for now my mother says… she only wants a grandchild. She says, stop this thing, man.”* **[P33, FGD01-01-04].** The young women however secretly continued using contraception.

#### Friends’ influence.

Participants reported how their friends’ bad experiences with the health delivery system negatively influenced their decision to use the health services. A participant narrated how her friend was ill-treated at a facility as she sought termination of pregnancy services. The friends then decided not to visit that health facility again. She argued that,

*“There’s no way you going to go back to that clinic. …, they did it to my friend, and as a friend, I would like to support, and when she said “I am no longer going there”, I said yes you’re no longer going, we go together. So I think it’s high time, our nurses, because they are older women, they should be taught how to treat us, they should be taught about our self-esteem. Some, we don’t come from like a group of women that makes us strong about our confidence.”* **[Participant P23, FGD 01-01-03].**

Friends’ negative perceptions of contraceptive use discouraged their peers’ continued use of services. The friends shared the myths they held against modern contraception use.

“...*With me, I think it’s the stigma within my peers, you, you’ll hear her saying, why are you preventing, why you giving this and that, you going to have a loose body, you going to lose your shape, you going to gain weight, you going to have a wet vj [vagina]”* **[Participant P16, FGD 01-01-01]**

### Community factors

Furthermore, participants explained how their communities had judgmental attitudes towards contraceptive use and believed that some contraceptives were similar to having an abortion. Some of the community members openly expressed their disapproval of young women, especially adolescents, using contraceptives.

*“In my community, they take it bad. I don’t know why because there is quite a high number of teenage pregnancies, but still, when you go for prevention they kind of make it feel as if you are doing a bad thing, or you are doing abortion in advance. They think… they usually say that you’re blocking the kids with injection.”* [**P11, FGD Participant**]

### Health system factors

We identified several health system-related factors which functioned as barriers to students’ use of health services. These include long waiting times, privacy, service provider attitudes, unfavourable opening hours, and low quality counselling services. Students also pointed out how the health service providers’ failure to provide counselling could lead to discontinued use of services.

#### Waiting time.

Long waiting times deterred some students from accessing contraceptive services. Most students reported waiting longer at PHCs and mobile clinics. A participant who thought that the waiting time at a PHC was too long said,

*“You know that when you are going to the clinic you have to get a lunch box or something because you are going to stay the whole day. That’s why a lot of girls don’t prevent because you know that if I want to prevent it means like the whole day I’m there*.” **[P15, FGD participant].**

#### Lack of privacy.

Lack of privacy at health services was one of the barriers students cited the most. There were different conceptualizations of privacy. Firstly, the students were uncomfortable with having their neighbours at home, knowing they were accessing SRH services. A student explained,

“… *at home, there is no privacy. Your neighbour, who is there for something else would probably know that you went to the clinic.”* [**P14, FGD Participant]**

Secondly, privacy was about not mixing with clients at the facility for different issues. The students felt they were not sick and did not want to mix with others. Mixing clients compromised privacy as some providers at PHCs would then go around the queue asking clients what services they intended to access on that particular day. A participant said,

*“You are there just for prevention and they just make you queue with everybody else and when you go there, you have to like… when they ask you what you here to do, you have to speak louder, I came, I came to prevent, contraceptive, what, I came to prevent. So everybody must know your business?”* **[Participant 14, FGD01-01-01]**

Participants believed that being in the same queue with other clients meant that it would take longer to get contraceptives. This was especially related to PHCs rather than campus clinics. In addition, students were sometimes asked to bring urine samples and had to pass through other clients while bringing the sample:

“…*there, there is no privacy when you have to urinate. You go to the outside toilet, and you come back with urine in front of people and stuff, yeah*…” **[Participant P17, FGD 01-01-01]**

#### Service provider attitudes.

Negative service provider attitudes were another barrier to contraceptive use among students. Students highlighted that providers would call each other when a client had missed an appointment date and address them in front of their peers. Students felt that such behaviour from service providers had a negative impact on their self-esteem and confidence. A student explained:

*“… when you missed your date, maybe you skipped by one month, you get stigma and then they’re so rude to a point that they call one another, “come see, come see, number 23, come see this child, but sis what’s wrong” Then imagine in front of your peers they do that to you, they ruin your self-esteem, and your confidence.”* [**P23, FGD 01-01-03 Participant**].

#### Unfavorable opening days and times.

Some students highlighted that they discontinued contraception because they could not access services at their local facility during their convenient times. One student reported that in addition to waiting long hours, she failed to access modern contraceptives from the same facility, which does not provide contraceptive services on Fridays and weekends. She said,

“…*in my local clinic … you take the whole day and then the other issue is that you cannot go on Friday, or Saturdays because they don’t work with preventions on Saturdays like at all. They require you to come during the week, and then when I was preventing, I was in high school then, so I did not have time, so it’s either it’s the weekend or nothing, that’s why I stopped preventing, because in, in grade 10 and 11 it was fine because I had time, in grade 12 I did not have time all.”* **[P15, FGD01-01-01 participant]**

#### Low quality counselling services.

Participants reported that they did not receive adequate counselling services. They felt the providers did not have enough time to explain contraceptive use and possible side effects. Students explained that the nurses forgot to inform them about what to expect, for example, gaining weight and guidelines on how to eat so that the young women would not gain excess weight.

“*… sometimes the way the nurses are, they don’t take their time to explain, they forget to tell you that yes, you will gain weight, but your responsibility is to eat well. Eat healthy food when you feel that you are hungry, because you will be hungry often, but eat healthy. Have fruits in your room, have snacks that are…peanuts, that are healthy.*” [**P23, FGD Participant]**

#### Being turned away from health facilities.

Students are a mobile population. Some students reported being turned away from PHCs in their home areas as they were asked to bring small notebooks which would be used as medical records that the home facilities recognised. However, when the semester break passed, they could not continue using them as they were not accepted at campus clinics and other facilities outside their home clinics. In addition, students with campus clinic cards or any institutional emblem or who mentioned that they were studying at the named institution known to have a campus clinic were turned away by nurses at a nearby PHC. The providers often told them they were using the facility’s medical supplies. Some students then resorted to copying information from campus cards and writing it on the books accepted by some PHCs, including the dates they were supposed to present at the facility, to avoid being turned away. Students, however, reported that some facility providers suspected they might have been writing in the books.

### Service providers promoted misinformation about some methods

Students reported being discouraged from using some contraceptive methods, like the implant, by service providers. Service providers told them that the implant could affect their future fertility. In an FGD participant said, “*Most of the time we’re advised that eish, do not use the…the implant because you do not have a child, so it might affect you in the future, that’s what we are told. Hence most of us are not using it.”* **[Participant P27, FGD01-01-03]**

### Students’ recommendations for long-acting PrEP

Participants suggested dealing with some of the family and community barriers to the uptake of long-acting PrEP. They suggested that there needs to be awareness raising directed at parents and community education, which they believed could normalize the use of PrEP and reduce stigma.

*“I think a way in which we can get parents to know about these things within the community is maybe at schools, a high school, especially like they host meetings and, and that’s where maybe a nurse will come in or somebody and, and just talk to parents, and inform them, so that parents are also… if maybe parents can come with their kids, boys and girls because boys also have their own stigma.”* [**P14, FGD participant]**

#### Quality of care.

Participants stressed the importance of quality of care. The issues of concern to be addressed if long-acting PrEP is to be rolled out successfully include negative provider attitudes, long waiting times, poor stock management, and lack of privacy. A participant noted that,

*“…what I think could be improved is the quality of the service in terms of how they speak to young people.”* **[03-01-05, IDI participant, 20 years].**

In addition, students made suggestions for improving planning, particularly related to stock management. “*I think they can find statistics for people around there so that when they render services, they know how many people there and how many people need those services.*” **[01-01-03, IDI participant 21 years]**

#### Shorten waiting times.

Reducing the waiting time at health facilities was one of the recommendations provided by students. A participant said, “*they should improve, and what is it called? They should improve the waiting period there.”* **[01-01-06, IDI participant, 19 years**]. Students suggested some ways which would help shorten waiting time, such as hiring more staff. One participant said, *“Let there be more staff around uhm…that is able to be at the disposal to help their patients, let there be someone to guide you in terms of where you supposed to go, let’s not all wait for the same person to help us out.”* **[IDI 01-01-14, participant, 19 years]**

In addition, students suggested that staff should take shorter breaks

*“… they must be fast because when they can go to break, they take like a long time to attend to patients. they must reduce it [breaks] to maybe one hour.”* [**03-01-06, IDI participant,20 years].**

#### Improve privacy.

Also, students stressed the importance of privacy in service delivery.

*“every clinic should have a youth zone so that young people can go like and actually have like enquire and get all the information that they need at their own private space.”* **[P14, FGD 01-01-01]**

#### Action to be taken about suggestion boxes.

Students had concerns on the way suggestion boxes were being handled at some PHCs. They recommended that healthcare facilities should ensure that suggestion boxes are not locked to allow clients to use them, “…*I think maybe the government must make sure that the suggesting box they don’t lock them because most of the clinics lock those suggestion boxes.”* **[01-01-01, IDI participant, 23-year-old].** In addition, students said that the suggestion box should be guarded by security personnel, citing examples of facilities with suggestion boxes which were closed to the public for use. Further, they suspected that some facilities threw the written complaints in dustbins.

“… *you see that lots of people are complaining about that clinic, but nothing is being improved to show that they remove those complaints, maybe they put in a dustbin, they don’t submit where they are supposed to***…” [01-01-01, IDI participant, 23-years**]

#### Younger nurses were more acceptable.

To improve service delivery, students suggested that older nurses should be replaced by younger nurses. They highlighted that the older nurses complained that they were old and wanted to go home earlier than the normal time. A participant said,

“… *the old nurses they complain that they are old, they want to knock off at half past three maybe before time or four o’clock. So, I think they must put at least youth there, maybe they will manage the time, cause [*because*] those ones they will tell you about their age.”* **[01-01-01, IDI participant, 23 years]**

## Discussion

This study explored the different barriers and facilitators to the uptake of current contraceptive offerings among students in tertiary institutions with the view to informing PrEP services. The study contributes to the body of research by adding the voices of young, mobile contraceptive users who are the same young women that PrEP services aim to reach. While other studies have identified facilitators and barriers to contraceptive use, this study was done in the context of HIV PrEP to inform its delivery with the consideration of integrating contraceptive and PrEP services at primary health care services and campus clinics. Applying the social-ecological framework to organize the findings from the study provided insights into facilitators and barriers to service utilization at different levels. Individual-level barriers in the form of side effects such as headaches were common to both contraception and PrEP hence for the successful delivery of PrEP, proper management of side effects should be ensured to avoid early method discontinuation. In addition, health system-level barriers like negative health provider attitudes were the major barriers to using current contraceptive offerings among students. While previous studies have also identified negative health service provider attitudes as a barrier to service use [11,14], in our study participants faced unique challenges as students. As they are a mobile population, moving between their hometown and the tertiary institution. We found that some barriers to accessing contraception arise as the primary health care clinics in their home areas fail to cater for those who usually receive services elsewhere. The same challenge is likely to arise for PrEP service access.

Our findings on individual barriers such as side effects are similar to reports of young women who had ever used oral PrEP which led to discontinuation due to the perceived severity of side effects [23,24]. Side effects if left unchecked could ultimately limit choices. Headaches are a common side effect of both contraception and PrEP [25]. Since our participants were students, headaches also affected their daily activities, such as studying, which influenced their decision to discontinue the methods. Previous studies have highlighted how service providers play a critical role in discussing and managing side effects [26]. Our study, however, found that providers did not adequately provide counselling and information on potential side effects. Investing more time discussing possible side effects would be important for the sustained use of PrEP. Providers should consider prescribing analgesia for headaches when offering both contraceptive and PrEP services.

Family, especially mothers, play a crucial role in contraceptive uptake and continued use. Our findings align with what was found in previous studies where mothers either encouraged or discouraged contraceptive use. Mothers facilitated young women’s access to contraceptives by providing information concerning the available methods on the market and guiding their daughters on which methods would work for them. The interesting contribution made by mothers in our study was the practical support they provided to their daughters, for example, after missing appointments, they could give them money to access services through a pharmacy. Also, participants reported being dropped at the health facilities by their mothers to get contraceptives. In addition, mothers played a facilitating role through monitoring and ensuring that their daughters attended appointments. However, in other studies, mothers or caregivers have shown reluctance to communicate to their daughters about contraception or PrEP due to lack of knowledge or socio-cultural norms around sexuality education including the fear that such communication would promote sexual behavior [27,28]. Mothers or other caregivers will need to be engaged in all these aspects to ensure that they do not hinder successful PrEP delivery. It will be, therefore, crucial to involve mothers in PrEP programmes, especially targeting the promotion of PrEP to families and communities as their support could facilitate young women initiating and adhering to PrEP.

Intimate partners are key influences for contraception use. In some instances, participants reported that they received partner support while in other cases partners were barriers to continuing contraception. The barriers included concerns from some partners about side effects and infertility. The myth that using contraception causes infertility is not unique to this study setting but has been reported to dominate the sub-Saharan and West African populations [29] Also, the disruption of sexual activities affected the male partners. For example, side effects reported by contraceptive pill users such as vaginal wetness and urinary tract infections (UTIs) affected intimacy and male partners then started to complain and discouraged continued use. Vaginal discharge, itchiness, headaches and UTIs are some of the reported side effects associated with the DVR [30]. As new long-acting PrEP methods such as the ring are becoming available, it will be essential to ensure that the male partners have information about the benefits and side effects. If young women support the idea, they could be involved in the counselling and in discussing how to manage the side effects. It will be also important to ensure that potential side effects are manageable, to limit the early discontinuation of contraceptives or PrEP.

Young women’s agency can be a facilitator of contraceptive use. Women’s agency in this context refers to their ability to define goals and act on them [31] using diverse strategies such as negotiation, deception, manipulation, resistance and subversion [32]. While a few participants reported discontinuing contraception due to partner influence, most young women enacted their agency and continued taking contraception secretly. Some scholars have argued that male partners can obstruct use which can be linked to discontinuation and unplanned pregnancies [33]. Other studies have however reported how agency has been often constrained by partner, family demands and uncertainty [34]. Some participants opted for non-disclosure of hormonal contraception in order to ensure the continued use of condoms for the prevention of HIV and sexually transmitted infection. Most students also had similar fears about disclosing PrEP use to their partners. Covert use of contraception or PrEP while demonstrating young women’s agency may lead to discontinuation in the longer term as the it does not address partner communication and negotiation skills [33]. However, covert use has also been viewed as reflecting higher levels of agency [35]. It will be essential to empower young women with information on long-acting PrEP and ensure they have the agency to overcome the barriers that may emanate from the partners.

### Heath service factors

#### Waiting time.

We found that waiting time plays a vital role in service utilisation. While a similar finding has been reported in other studies [36], our study population were university students hence time is essential in their lives as they must attend lectures and study. Long waiting times imply that they are sacrificing study time including attending lectures. However, shorter waiting times reported at campus facilities could be because such clinics offer services exclusively to students, whereas home clinics are open to the public. In addition, campus and mobile clinic staff is trained to provide youth-friendly services, so even though the waiting time is long, the young women are comfortable with waiting. There is need to test strategies to shorten waiting times especially at public health facilities. This is relevant across different Southern and East African countries faced with similar health systems challenges, especially related to adequate human resources.

#### Privacy.

Our study found that students value privacy in service provision. While previous studies had reported that lack of privacy was a concern [37], our study showed that privacy was especially a concern when students accessed services from PHCs. They explained different dimensions of privacy, including not being seen by older people from their communities while accessing services at PHCs. A similar barrier at the community level was also identified in a different setting [37]. Students praised campus clinics for providing privacy and confidentiality. Students accessing campus clinics had the privilege of accessing services only with their peers. These students’ expectations of privacy may therefore be higher than their peers who only use PHCs. It will be important for PHCs to invest in youth zones which are youth dedicated areas at public health facilities.

### Provider attitudes

Service providers’ attitudes were raised as a concern, for example, students being shamed in front of their peers for a missed appointment. Participants reported that this affected their confidence and self-esteem. Providers, especially at public health facilities, should be trained to provide services in a non-judgmental way. How students are treated as they receive the service matters to them. This will be important to consider as long-acting PrEP products are delivered. The challenge of healthcare provider attitudes is not unique to the South African context but occurs across the sub-Saharan African region [38,39]

Students also reported how providers misinformed them especially about the implant stating that it caused infertility. A similar finding has been reported elsewhere [40,41]. Misinformation limits choices and discourages the initiation or continuation of methods. As more PrEP options are closer to being introduced, it will be important for providers to provide unbiased choice counselling so that young women can choose the method that works better for them and switch where necessary. The training of healthcare providers and ongoing supervision will be essential to ensure that the information provided to young women is accurate. In addition, contraceptive and PrEP counselling should be more responsive to young women’s dynamic lives especially their relationship statuses, shifting preferences, values, perceptions, beliefs and experiences as these shape their decision-making compared to biomedical advice [42]. Young women’s decision-making is therefore shaped by various structural, contextual, interpersonal coupled with the changing values, preferences and needs [42]. The provision of both contraceptive and PrEP counselling based on the reproductive justice framework is imperative in order to ensure equitable access to pregnancy prevention and HIV prevention methods for young women.

Students’ mobility negatively impacts access to health services and continuity of care. Access to health services was often interrupted during semester breaks as students were sometimes turned away from some health facilities as they needed to have health record booklets different from those used by campus clinics. It is crucial to develop a centralised database and acceptable referral arrangements to ensure continuity of care as students’ transition between campus and facilities in their home areas. In South Africa, electronic health systems have been produced by different vendors which have however failed to communicate with each other as they were built using different underlying database architectures [43]. While electronic systems have been implemented in some areas, more than half of public health systems in South Africa were still using paper-based records [43]. Attempts have been made to digitize the health system in African countries like Ghana and Malawi failed due to the absence of government support, supporting infrastructure and a reliable electricity supply among other reasons [43–45]. As long-acting PrEP methods become available, it will be necessary for campus and public facilities to accept each other’s health records, referrals, and prescriptions to enable mobile patients to still benefit from any PHC. Linking the services will also ensure that students do not mess with their health records trying to copy what is on the campus clinic cards onto the writing books. Also, students will also complete their studies at some point and need to easily fit into the health systems outside the campus; hence, services need to be fully linked while these young women are still students. This will also be important in preparation for the implementation of national health insurance.

## Study strengths and limitations

Data were collected using multiple methods including FGDs and IDIs which allowed for triangulation of findings. Young women were very open about their experiences of contraceptive services and discussed their issues openly. Despite the strengths, we recognise the limitations of our study. Firstly, the focus on students in tertiary institutions alone leaves out the views of other young women which are also crucial in informing service delivery. The study also only reported on students’ views as we did not interview healthcare providers. Future studies should also look at the views of healthcare providers. In addition, social desirability bias could have affected our findings as we solely relied on students’ responses to questions.

## Conclusion

Facilitators and barriers to contraceptive use occur at different levels. A multipronged approach is therefore essential to ensure continued use of both PrEP and contraceptive methods. There are striking similarities between the facilitators and barriers to accessing and maintaining contraceptive use and PrEP. As our findings show that there are side effects common to both contraceptives and PrEP use, this offers an opportunity to improve counselling and management of the side effects to promote their effective use. Also, lessons learned from either contraception or PrEP could strengthen both services. As both contraception and PrEP fall under the same umbrella of SRH services, according to UNAIDS reports on targets for 2025, there is a strong case for integrating services. In addition, the challenges experienced by young university students point to a need for health systems to ensure continuity of care through catering for mobile people, such as students who change places where they access contraception during the term, on vacation and when they finally complete their studies and can no longer access the campus clinics

## Recommendations for PrEP

PrEP is being rolled out in many high HIV prevalence settings, particularly in Southern and East Africa which are characterized by a generalized epidemic. There are several recommendations which can be drawn from this study which would be relevant in other settings where PrEP is being rolled out. Firstly, it will be important to invest more in ongoing provider training, especially on the management of side effects. This will address barriers for PrEP use at individual level. Providers should spend more time discussing potential side effects and prescribing analgesics to manage headaches, if required. In addition, it will be important to offer remedies for nausea to support continued use of PrEP should young women struggle with these side effects. A previous study reported that about 40% of the participants discontinued oral PrEP due to nausea [4]. Managing side effects therefore enables continued use of methods. Also, contraceptive and PrEP counselling should be more responsive to young women’s lives, relationship status, perceptions, beliefs and embodied experiences as these play a key role in their decision-making.

At a relationship and family level, it will be important to provide PrEP information to partners and parents as they play an essential role in the continued use of contraception. Partners and parents have the potential to play the same key role in PrEP use. Studies conducted elsewhere have reported how male partners played a key role, especially in reminding and encouraging their partners to take PrEP, which enabled continued PrEP use [46,47].

There is also a need to improve the quality of service delivery. Many health-system factors still need to be addressed to improve service delivery. Service provider attitudes remain a crucial barrier to the utilisation of contraceptive services and potentially PrEP services. At the health system levels, more providers need to be trained to provide youth friendly services so they can encourage young women to access services through their positive, non-judgmental attitudes. Providing contraceptives for free does not translate to the uptake of services hence there is need to improve the quality of services.

Waiting times need to be shorter, especially for young women who are studying. This would facilitate continued access to SRH services including, for long-acting PrEP. Long waiting times are a key barrier to client satisfaction and quality of service [16] and need to be addressed through finding innovative ways of shortening waiting times. Opening hours need to be addressed as some students only get time after their classes when some PHCs would have closed already. For continued use of services, it will be important to consider offering services both during weekends and after hours as unfavorable working hours discourage service utilization. Proper stock management will also help gain the trust of young women in the health system.

Since students are a mobile population, it will be crucial to have electronic health records. A centralised database will ensure a smooth transition between different service delivery points. This will likely result in continuation of care as students’ transition from their home provinces to the tertiary sites and vice versa so that users do not end up tempering health facility records trying to create a clinic card or booklet acceptable to the facility they are visiting. Also, students will eventually complete their studies, but the need to prevent HIV remains; hence, it is important to integrate the services by creating a centralised database.
